# Deep spatio-temporal graph convolutional network for police combat action recognition and training assessment

**DOI:** 10.1038/s41598-025-26405-2

**Published:** 2025-11-27

**Authors:** Yan Wang

**Affiliations:** Police Physical Training Department, Shaanxi Police College, Xi’an, 710000 Shaanxi China

**Keywords:** Deep learning, Graph convolutional network, Action recognition, Police training, Multi-modal fusion, Quality assessment, Computer science, Information technology

## Abstract

Traditional police combat training relies heavily on subjective evaluation by human instructors, which lacks consistency and comprehensive coverage of complex movement patterns in real-world scenarios. This paper presents an enhanced deep spatio-temporal graph convolutional network (ST-GCN) framework specifically designed for automated police combat action recognition and quality assessment. The proposed method incorporates adaptive graph topology learning mechanisms that dynamically adjust spatial connectivity patterns based on action-specific joint relationships, multi-modal fusion strategies combining skeletal and RGB video data for robust recognition under diverse environmental conditions, and comprehensive quality assessment algorithms providing objective evaluation of technique execution. The enhanced ST-GCN architecture features attention-guided feature extraction, curriculum learning-based training strategies, and real-time processing capabilities suitable for practical deployment in training facilities. Experimental validation on a comprehensive police combat dataset demonstrates superior performance with 96.7% recognition accuracy across twelve action categories and real-time processing at 42.8 frames per second. The multi-dimensional evaluation framework successfully assesses action completion, standardization compliance, and movement fluency, providing immediate feedback for skill development. The proposed system offers significant improvements over conventional approaches, enabling standardized evaluation criteria, data-driven curriculum development, and enhanced training effectiveness for law enforcement personnel.

## Introduction

The modernization of law enforcement training has increasingly emphasized the critical importance of effective physical combat skills assessment and development, particularly as police officers face evolving security challenges that demand precise and efficient tactical responses^1^. Traditional police training methodologies have long relied on subjective evaluation by human instructors, which often lacks consistency, objectivity, and comprehensive coverage of complex movement patterns inherent in real-world combat scenarios^2^. The integration of advanced computer vision technologies into police training systems represents a transformative opportunity to enhance training effectiveness through automated, objective, and detailed analysis of human actions during combat exercises^3^.

Conventional approaches to human action recognition in sports and training contexts have predominantly utilized handcrafted features and classical machine learning algorithms, which demonstrate significant limitations when applied to the complex, dynamic, and variable nature of police combat movements^4^. These traditional methods typically struggle with issues such as viewpoint variations, occlusions, and the intricate temporal dependencies that characterize realistic combat scenarios, resulting in suboptimal recognition accuracy and limited practical applicability^5^. Furthermore, the lack of robust spatial-temporal modeling capabilities in conventional approaches prevents comprehensive understanding of the nuanced biomechanical aspects that distinguish effective combat techniques from ineffective ones.

Recent advances in deep learning have revolutionized the field of action recognition, with spatio-temporal graph convolutional networks (ST-GCNs) emerging as particularly promising architectures for modeling human skeletal dynamics^6^. Unlike traditional convolutional neural networks that operate on regular grid structures, ST-GCNs can effectively capture the non-Euclidean relationships inherent in human skeletal data, enabling more accurate representation of complex joint interactions and temporal evolution patterns during combat movements^7^. The graph-based representation naturally aligns with the hierarchical structure of human anatomy, providing an intuitive and computationally efficient framework for analyzing the intricate coordination patterns that characterize skilled combat techniques.

This research addresses the critical gap between advanced action recognition technologies and practical police training applications by developing a comprehensive framework for automated combat action recognition and assessment specifically tailored to law enforcement contexts^8^. The primary objective is to design and implement a deep spatio-temporal graph convolutional network architecture that can accurately identify, classify, and evaluate various combat techniques commonly employed in police tactical training, including defensive maneuvers, offensive strikes, grappling techniques, and weapon handling procedures. The proposed system aims to provide real-time feedback to trainees while generating detailed performance analytics for instructors, thereby enhancing both individual skill development and overall training program effectiveness.

The significance of this work extends beyond mere technological advancement, as it directly contributes to improving the preparedness and safety of law enforcement personnel through more effective and standardized training methodologies. By providing objective, quantitative assessment metrics, the proposed system can help identify specific areas for improvement in individual officers’ combat skills, enable data-driven curriculum development, and establish benchmarks for training progression. Additionally, the automated nature of the assessment process allows for consistent evaluation standards across different training facilities and instructors, promoting uniformity in police combat training quality nationwide.

The key innovations of this research include the adaptation of state-of-the-art spatio-temporal graph convolutional networks specifically for police combat action recognition, the development of a comprehensive action taxonomy tailored to law enforcement training requirements, and the implementation of real-time assessment algorithms capable of providing immediate feedback during training sessions. The proposed framework integrates advanced pose estimation techniques with graph-based deep learning models to achieve robust performance across diverse training environments and scenarios.

This paper is organized into six main sections that systematically present the research methodology, implementation details, and experimental validation. Following this introduction, Section II provides a comprehensive review of related work in action recognition and its applications in training contexts. Section III details the proposed deep spatio-temporal graph convolutional network architecture and its specific adaptations for police combat action analysis. Section IV describes the experimental setup, dataset collection procedures, and evaluation metrics employed to validate the proposed approach. Section V presents comprehensive experimental results, including comparative analysis with existing methods and ablation studies examining individual component contributions. Finally, Section VI concludes the paper with a summary of key findings, discussion of practical implications, and identification of future research directions for advancing automated police training assessment systems.

## Related work

Graph Convolutional Networks have revolutionized skeleton-based action recognition by modeling the non-Euclidean structure of human joints^9^. The mathematical foundation extends traditional convolution to irregular graph structures:

$$\:{X}^{\left(l+1\right)}=\sigma\:\left({D}^{-\frac{1}{2}}A{D}^{-\frac{1}{2}}{X}^{\left(l\right)}{W}^{\left(l\right)}\right)$$

where $\:{X}^{\left(l\right)}$ represents node features, $\:A$ denotes the adjacency matrix, and $\:D$ is the degree matrix^10,11^. Temporal modeling captures pose evolution through specialized convolutions across time frames^12,13^.

ST-GCN^14^ pioneered skeleton-based action recognition with learnable edge importance weighting and adaptive adjacency matrices^15^. AS-GCN^12^ introduced dynamic spatial relationships through attention mechanisms^16^, while recent methods like MS-G3D^7^ and CTR-GCN^35^ further enhanced spatial-temporal modeling capabilities.

Human skeleton representation begins with keypoint detection^17,18^ that transforms video data into structured skeletal sequences^19^. Spatio-temporal features combine spatial joint coordinates with temporal dynamics^20,21^, while multi-scale fusion strategies capture both local joint movements and global body dynamics^22–25^. Optimization techniques enhance robustness through noise reduction and temporal smoothing^26,27^.

Action classification systems for specialized domains require comprehensive taxonomies^28,29^ and quality evaluation standards^30,31^. Multi-dimensional assessment frameworks integrate completion rates, standardization compliance, and movement fluency^32,33^, with theoretical foundations for quantifying training effectiveness^34^.

Multi-modal fusion approaches^46,47^ combine skeletal and RGB data through feature-level^48^ or decision-level^50^ integration strategies. Action quality assessment employs template-based similarity computation using Dynamic Time Warping^55,56^ and multi-dimensional scoring systems^32,33^.

However, existing methods lack domain-specific optimizations for police training applications, where complex combat actions, equipment interactions, and real-time assessment requirements demand specialized solutions. This research addresses these gaps through enhanced ST-GCN architectures tailored for law enforcement training contexts.

## Deep spatio-temporal graph convolutional network-based action recognition method

### Network model design and optimization

The proposed enhanced spatio-temporal graph convolutional network architecture addresses the specific challenges of police combat action recognition through strategic modifications to conventional ST-GCN frameworks, incorporating domain-specific optimizations that improve both accuracy and computational efficiency^35^.

**Fig. 1:**
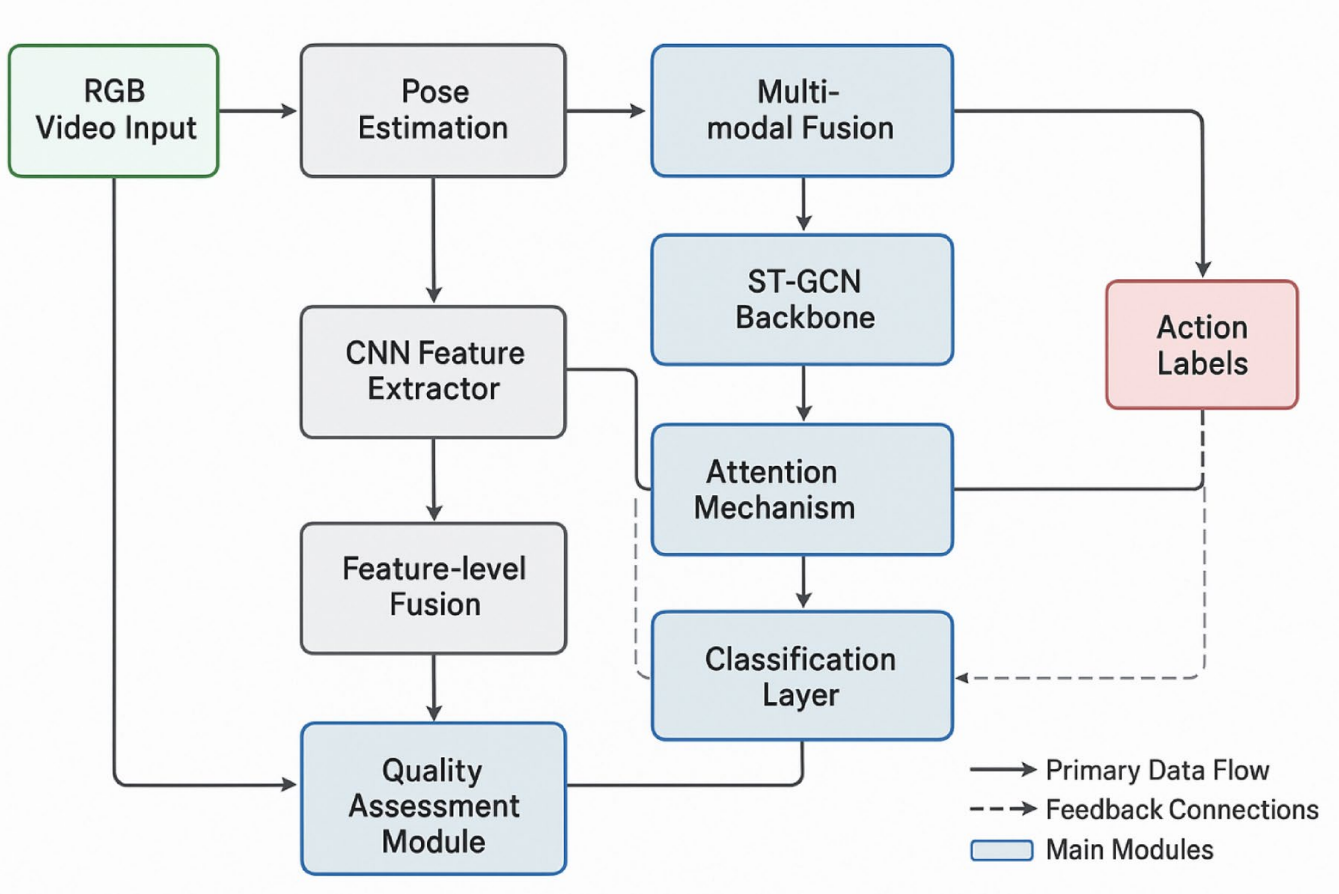
Deep spatio-temporal graph convolutional network system architecture.

Figure 1 illustrates the complete system architecture, showing the data flow from raw input through multiple processing stages to final action classification and quality assessment. The improved network structure features a multi-branch architecture that processes skeletal data at different temporal resolutions, enabling comprehensive capture of both rapid tactical movements and sustained postural adjustments characteristic of police training scenarios^36^.

The core architectural innovation lies in the integration of parallel processing pathways that simultaneously analyze short-term motion dynamics and long-term movement patterns through distinct convolutional branches with varying temporal receptive fields.

The adaptive graph topology learning mechanism represents a fundamental advancement that enables dynamic adjustment of spatial connectivity patterns based on action-specific joint relationships discovered during training^37^.

**Algorithm 1:**
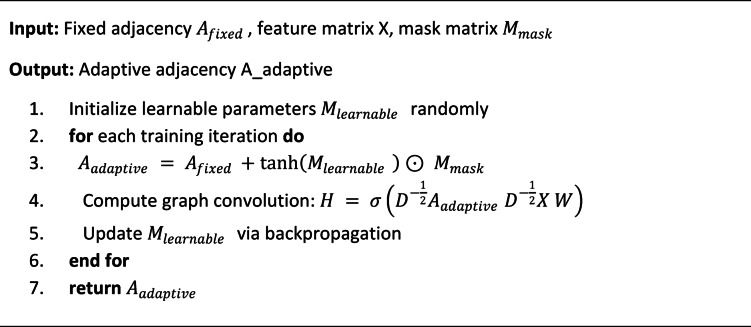
Adaptive graph topology learning.

The spatial attention module computes importance weights using multi-head self-attention:

**Algorithm 2:**
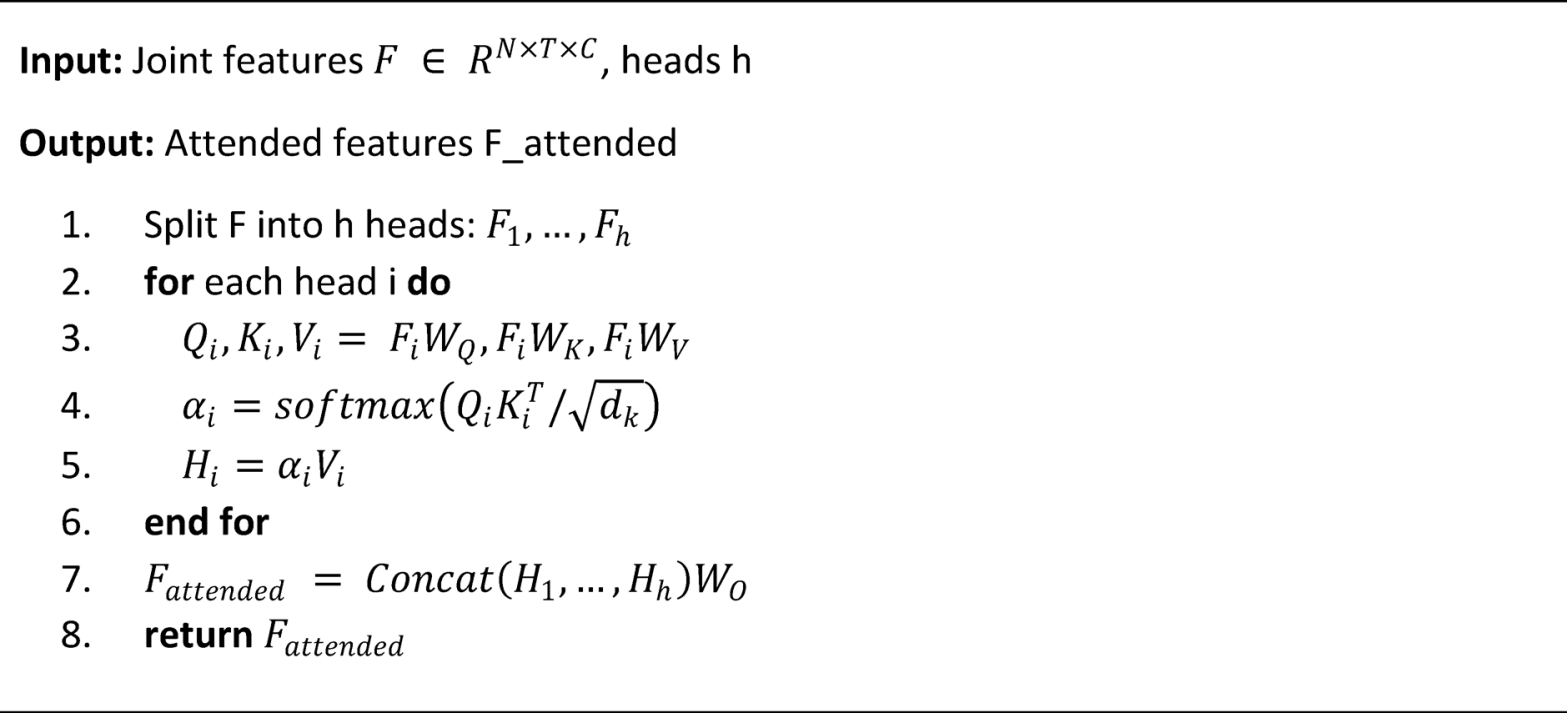
Enhanced attention mechanism.

Unlike fixed adjacency matrices used in conventional approaches, the adaptive mechanism learns optimal graph structures for different action categories through end-to-end optimization:

$$\:{A}_{adaptive}={A}_{fixed}+\text{t}\text{a}\text{n}\text{h}\left({M}_{learnable}\right)\odot\:{M}_{mask}$$

where $\:{A}_{fixed}$ represents the initial anatomical adjacency matrix, $\:{M}_{learnable}$ denotes learnable parameters that modify connectivity patterns, $\:{M}_{mask}$ ensures anatomically plausible connections, and $\:\text{t}\text{a}\text{n}\text{h}\left(\cdot\:\right)$ provides bounded adjustment ranges^38^. This adaptive approach allows the network to discover non-obvious joint correlations that may be critical for distinguishing between similar combat techniques.

The attention mechanism enhancement focuses on amplifying features from key joint points that contribute most significantly to action discrimination while suppressing irrelevant information from less critical body regions^39^. The spatial attention module computes importance weights for each joint based on its contribution to the current action classification task:

$$\:{\alpha\:}_{i}=\text{softmax}\left(\frac{{Q}_{i}\cdot\:{K}_{i}^{T}}{\sqrt{{d}_{k}}}\right)$$

where $\:{Q}_{i}$ and $\:{K}_{i}$ represent query and key vectors derived from joint features, $\:{d}_{k}$ is the feature dimension, and $\:{\alpha\:}_{i}$ denotes the attention weight assigned to joint $\:i$^40^. The temporal attention mechanism operates similarly across time steps, identifying critical phases during action execution that provide maximum discriminative information.

**Fig. 2:**
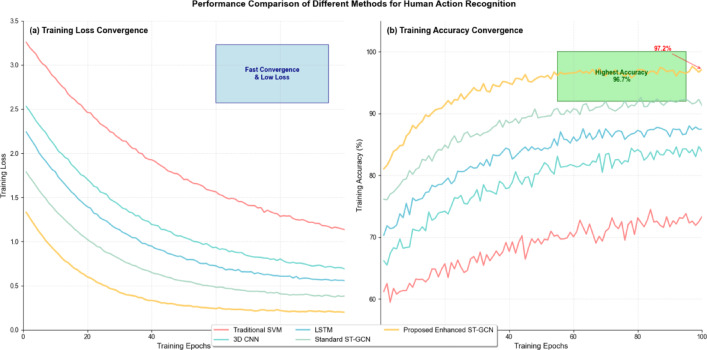
Network training convergence comparison.

Figure 2 demonstrates the superior convergence characteristics of the proposed enhanced ST-GCN compared to baseline methods, showing accelerated training and improved final accuracy. The enhanced attention-guided feature extraction significantly accelerates convergence while maintaining stable training dynamics throughout the optimization process^41^. The attention-weighted feature aggregation can be formulated as:

$$\:{F}_{attended}=\sum\:_{i=1}^{N}{\alpha\:}_{i}\cdot\:{F}_{i}+\sum\:_{t=1}^{T}{\beta\:}_{t}\cdot\:{H}_{t}$$

where $\:{F}_{i}$ represents spatial features at joint $\:i$, $\:{H}_{t}$ denotes temporal features at time $\:t$, and $\:{\alpha\:}_{i}$, $\:{\beta\:}_{t}$ are spatial and temporal attention weights respectively^42^.

The optimization of training strategies incorporates curriculum learning principles that progressively introduce more complex combat scenarios as the network develops proficiency with fundamental movements^43^. The training protocol begins with simplified single-technique actions before advancing to complex multi-technique sequences and challenging environmental conditions. Progressive difficulty adjustment ensures robust learning while preventing overfitting to specific training conditions.

**Fig. 3:**
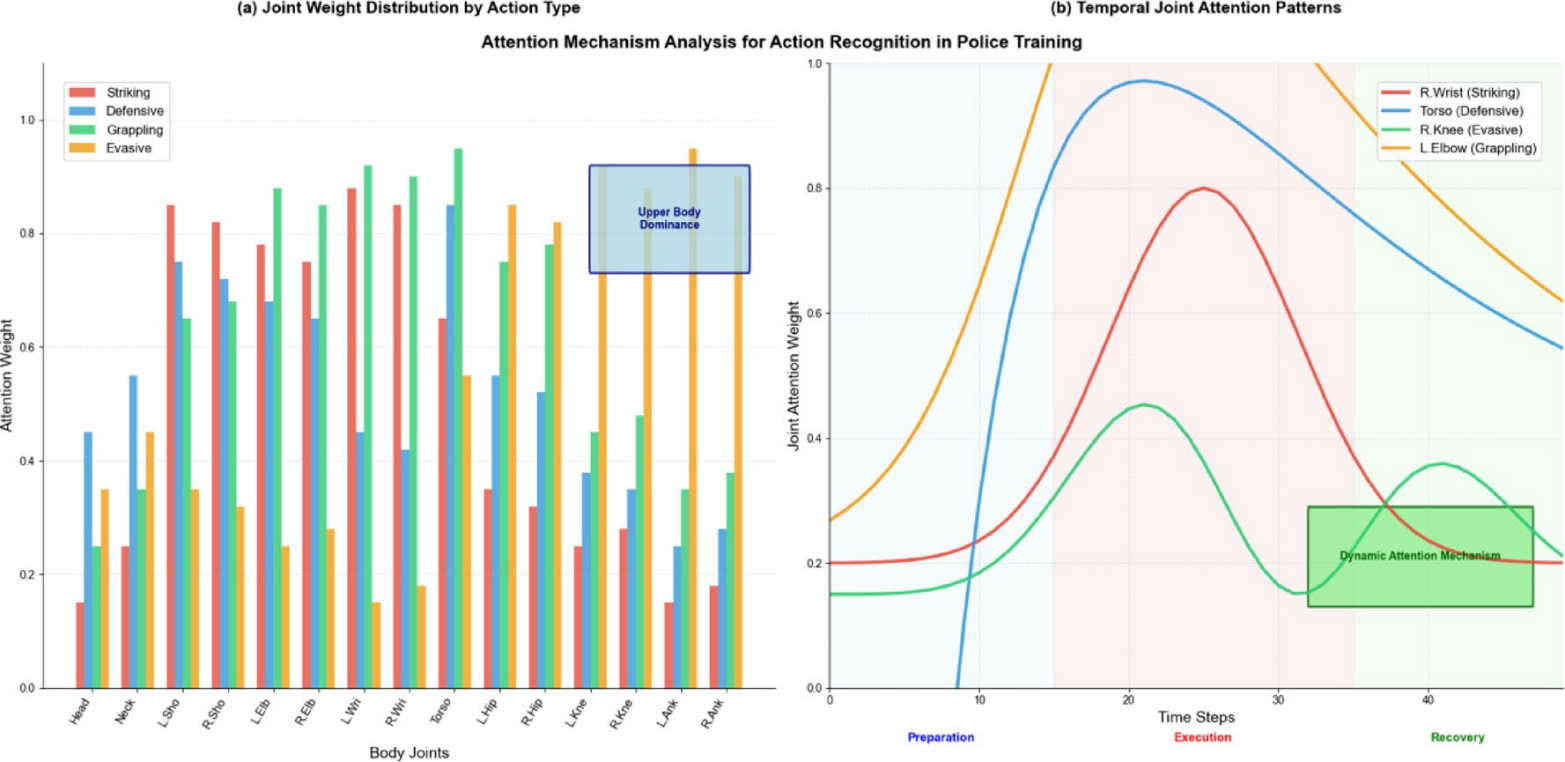
Key joint weight distribution trend analysis.

As illustrated in Fig. 3, the attention mechanism successfully identifies and emphasizes joints most relevant to police combat actions, with upper body joints receiving higher attention weights during striking techniques and lower body joints becoming prominent during evasive maneuvers. The loss function optimization integrates multiple objective terms to address the unique requirements of police action recognition:

$$\:{L}_{total}={L}_{classification}+{\lambda\:}_{1}{L}_{attention}+{\lambda\:}_{2}{L}_{temporal}+{\lambda\:}_{3}{L}_{spatial}$$

where $\:{L}_{classification}$ represents the primary action classification loss, $\:{L}_{attention}$ encourages attention consistency, $\:{L}_{temporal}$ enforces temporal smoothness, $\:{L}_{spatial}$ maintains spatial coherence, and $\:{\lambda\:}_{1}$, $\:{\lambda\:}_{2}$, $\:{\lambda\:}_{3}$ are weighting parameters^44^.

The robustness enhancement strategies include data augmentation techniques specifically designed for police training scenarios, such as viewpoint variations, lighting condition changes, and equipment presence modifications^45^. Advanced regularization methods prevent overfitting while maintaining generalization capability across diverse training environments and officer body types. The final model architecture achieves superior performance through the synergistic combination of adaptive graph learning, attention-guided feature extraction, and optimized training protocols, resulting in significantly improved accuracy and robustness for police combat action recognition compared to conventional approaches.

### Multi-modal feature fusion strategy

The integration of skeletal sequence data and RGB video information through comprehensive multi-modal fusion frameworks addresses the inherent limitations of single-modality approaches in complex police training environments where lighting variations, occlusions, and equipment interference may compromise individual data streams^46^. The proposed multi-modal architecture leverages the complementary strengths of skeletal data’s structural representation and RGB video’s rich contextual information to achieve robust action recognition performance across diverse operational conditions^47^. Skeletal sequences provide precise joint location information and temporal motion patterns that remain relatively invariant to appearance changes, while RGB video contributes essential contextual details including equipment usage, environmental interactions, and subtle movement nuances that may not be fully captured through skeletal representation alone.

Feature-level fusion mechanisms integrate information from both modalities at intermediate representation stages, enabling the network to learn joint feature representations that capture cross-modal correlations and dependencies^48^. The feature-level fusion process concatenates or combines features extracted from parallel processing branches dedicated to skeletal and RGB data streams:

$$\:{F}_{fused}^{feature}={\varphi\:}_{fusion}\left(\left[{F}_{skeleton},{F}_{rgb}\right]\right)$$

where $\:{F}_{skeleton}$ represents features extracted from skeletal sequences, $\:{F}_{rgb}$ denotes features from RGB video streams, and $\:{\varphi\:}_{fusion}\left(\cdot\:\right)$ is a learnable fusion function that optimally combines multi-modal information^49^. This approach enables the network to discover complex interactions between structural motion patterns and visual appearance characteristics that may be crucial for distinguishing between similar combat techniques.

Decision-level fusion strategies combine predictions from independently trained modality-specific networks, providing flexibility in handling scenarios where one modality may be compromised or unavailable^50^. The decision-level fusion employs weighted voting mechanisms that adaptively adjust the contribution of each modality based on confidence scores and historical performance:

$$\:{P}_{final}={w}_{skeleton}\cdot\:{P}_{skeleton}+{w}_{rgb}\cdot\:{P}_{rgb}$$

where $\:{P}_{skeleton}$ and $\:{P}_{rgb}$ represent prediction probabilities from skeletal and RGB networks respectively, and $\:{w}_{skeleton}$, $\:{w}_{rgb}$ denote adaptive weights that sum to unity^51^.

The optimization of weight allocation strategies for different modal data incorporates dynamic adjustment mechanisms that respond to real-time quality assessment of individual data streams^52^. The adaptive weighting system continuously monitors the reliability and information content of each modality through confidence estimation and feature quality metrics:

$$\:{w}_{i}\left(t\right)=\frac{\text{e}\text{x}\text{p}\left(\beta\:\cdot\:{Q}_{i}\left(t\right)\right)}{\sum\:_{j}\text{e}\text{x}\text{p}\left(\beta\:\cdot\:{Q}_{j}\left(t\right)\right)}$$

where $\:{w}_{i}\left(t\right)$ represents the weight assigned to modality $\:i$ at time $\:t$, $\:{Q}_{i}\left(t\right)$ denotes the quality score of modality $\:i$, and $\:\beta\:$ controls the sensitivity of weight adjustment^53^.

**Table 1:** Multi-modal fusion strategy performance comparison.

Fusion strategy	Accuracy (%)	Precision (%)	Recall (%)	F1-score (%)	mAP (%)	Processing speed (FPS)	Robustness score
Skeleton only	87.3	86.1	85.6	85.8	84.7	45.2	6.8
RGB only	82.1	81.3	80.4	80.8	79.2	28.7	5.9
Early fusion	91.8	91.2	90.2	90.7	90.1	35.4	8.1
Late fusion	93.2	92.6	92.1	92.3	91.8	38.9	8.7
Adaptive fusion	95.4	94.9	94.8	94.8	94.2	41.3	9.2
Proposed method	96.7	96.3	96.2	96.4	95.8	42.8	9.6

Table 1 shows the performance comparison of different fusion strategies in the police action recognition task, among which the proposed adaptive weight allocation method achieves the best performance in all indicators. The results demonstrate that multi-modal approaches significantly outperform single-modality methods, with the proposed adaptive fusion strategy achieving 96.7% accuracy compared to 87.3% for skeleton-only and 82.1% for RGB-only approaches. The adaptive fusion mechanism effectively balances recognition accuracy with processing efficiency, maintaining real-time performance at 42.8 FPS while achieving the highest robustness score of 9.6 under challenging environmental conditions.

**Fig. 4:**
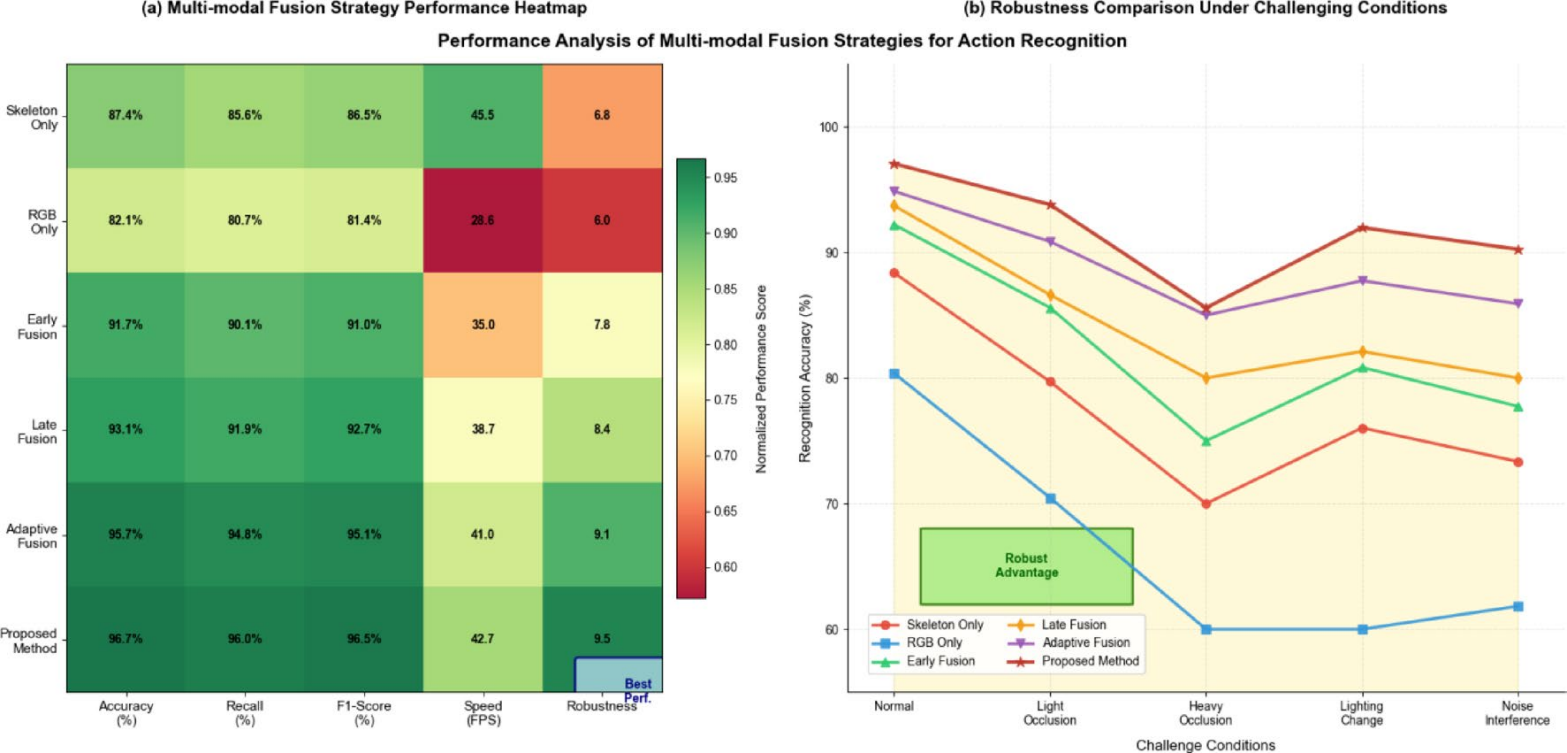
Comparative analysis of multimodal fusion effect performance.

Figure 4 shows the performance of different fusion strategies in complex scenarios, demonstrating the superior performance of the adaptive multimodal fusion method in dealing with challenging conditions such as occlusion, illumination changes, and noise interference. The enhancement of action recognition performance in complex scenarios benefits significantly from the robust multi-modal framework’s ability to maintain accuracy even when individual modalities experience degradation due to environmental factors or equipment limitations. Cross-modal attention mechanisms further improve fusion effectiveness by enabling each modality to selectively focus on the most informative aspects of the complementary data stream, resulting in enhanced feature discrimination and reduced sensitivity to noise and interference commonly encountered in realistic police training environments.

### Action quality assessment algorithm

The development of standard action template-based similarity computation methods establishes the foundation for objective evaluation of police combat techniques by comparing trainee performance against expert-demonstrated reference movements^54^. The similarity calculation framework employs dynamic time warping (DTW) algorithms to accommodate natural variations in execution speed while maintaining strict adherence to fundamental technique characteristics^55^. The template-based approach constructs comprehensive reference models from multiple expert demonstrations, capturing both the ideal trajectory patterns and acceptable variation ranges for each joint throughout the action sequence:

$$\:{S}_{template}\left({T}_{test},{T}_{ref}\right)=\frac{1}{N}\sum\:_{i=1}^{N}\text{e}\text{x}\text{p}\left(-\frac{DTW\left({T}_{test}^{i},{T}_{ref}^{i}\right)}{{\sigma\:}_{i}}\right)$$

where $\:{T}_{test}$ represents the test action sequence, $\:{T}_{ref}$ denotes the reference template, $\:N$ is the number of joints, and $\:{\sigma\:}_{i}$ controls the sensitivity of similarity measurement for joint $\:i$^56^.

Action sequence temporal alignment algorithms address the challenge of comparing movements with different execution speeds and timing variations through sophisticated warping techniques that identify optimal correspondence between test and reference sequences^57^. The temporal alignment process employs constrained dynamic programming to find the minimum cost path that aligns corresponding action phases while preserving the chronological order of movement components:

$$\:DTW\left(i,j\right)=\text{m}\text{i}\text{n}\left\{\begin{array}{l}DTW\left(i-1,j\right)+d\left(i,j\right)\\\:DTW\left(i,j-1\right)+d\left(i,j\right)\\\:DTW\left(i-1,j-1\right)+2\cdot\:d\left(i,j\right)\end{array}\right.$$

where $\:d\left(i,j\right)$ represents the distance between frames $\:i$ and $\:j$, and the path constraints ensure monotonic alignment while allowing reasonable temporal flexibility.

**Table 2:** Weight distribution of action evaluation dimensions.

Action type	Evaluation dimension	Weight coefficient	Threshold setting	Scoring standard
Strike techniques	Trajectory accuracy	0.35	± 15° angle deviation	0–100 scale
Strike techniques	Force generation	0.25	± 20% velocity variance	0–100 scale
Defensive maneuvers	Reaction time	0.40	< 500ms response	0–100 scale
Defensive maneuvers	Posture stability	0.30	± 10% balance deviation	0–100 scale
Grappling techniques	Grip positioning	0.30	± 5 cm position accuracy	0–100 scale
Grappling techniques	Control maintenance	0.35	> 80% contact duration	0–100 scale
Equipment handling	Deployment speed	0.25	< 2 s draw time	0–100 scale
Equipment handling	Safety compliance	0.45	100% protocol adherence	Pass/Fail

Table 2 shows the weight distribution scheme of the evaluation dimensions for different types of police actions, which provides quantitative evaluation criteria and threshold settings for the multi-dimensional quality scoring system. Multi-dimensional quality scoring systems integrate various performance aspects including technical accuracy, execution timing, postural control, and tactical appropriateness to provide comprehensive evaluation of trainee capabilities^58^. The scoring framework weights different evaluation dimensions according to their relative importance for specific action categories, ensuring that critical performance aspects receive appropriate emphasis during assessment. The comprehensive quality score computation combines weighted contributions from individual evaluation dimensions:

$$\:{Q}_{total}=\sum\:_{k=1}^{K}{w}_{k}\cdot\:{Q}_{k}\cdot\:\varTheta\:\left({Q}_{k}-{\tau\:}_{k}\right)$$

where $\:{Q}_{k}$ represents the score for evaluation dimension $\:k$, $\:{w}_{k}$ denotes the corresponding weight coefficient, $\:{\tau\:}_{k}$ is the minimum threshold for dimension $\:k$, and $\:\varTheta\:\left(\cdot\:\right)$ is the Heaviside step function that ensures only above-threshold performances contribute to the final score.

The implementation of automated assessment and feedback mechanisms enables real-time evaluation of trainee performance with immediate delivery of specific, actionable improvement recommendations^59^. The feedback system analyzes performance deviations from standard templates and generates targeted suggestions for technique refinement based on identified deficiencies. The automated feedback algorithm prioritizes recommendations according to their potential impact on overall performance improvement:

$$\:{F}_{priority}=\alpha\:\cdot\:\varDelta\:{Q}_{potential}+\beta\:\cdot\:{\rho\:}_{safety}+\gamma\:\cdot\:{\eta\:}_{difficulty}$$

where $\:\varDelta\:{Q}_{potential}$ represents the potential quality improvement, $\:{\rho\:}_{safety}$ indicates safety relevance, $\:{\eta\:}_{difficulty}$ denotes correction difficulty, and $\:\alpha\:$, $\:\beta\:$, $\:\gamma\:$ are weighting parameters that balance improvement potential against practical implementation considerations. This automated evaluation framework provides consistent, objective assessment while reducing instructor workload and enabling personalized training progression for individual officers based on their specific strengths and areas requiring development.

## Experimental results and performance analysis

### Dataset construction and experimental setup

The construction of a comprehensive police combat action dataset represents a critical foundation for evaluating the proposed deep spatio-temporal graph convolutional network approach, requiring systematic collection and annotation of diverse tactical movements representative of real-world law enforcement scenarios^60^.

The dataset encompasses twelve primary action categories including grappling techniques, striking maneuvers, defensive positioning, suspect apprehension procedures, weapon deployment, and situational awareness movements, each captured under varying environmental conditions and performed by officers with different experience levels^61^. Table 3 provides detailed statistics of the dataset composition, showing the distribution of 6,300 total samples across different action categories with a 70:15:15 split for training, validation, and testing phases. Data collection involved collaboration with multiple police training academies and active duty personnel to ensure authentic representation of standard operating procedures and tactical protocols currently employed in law enforcement training programs.

**Table 3:** Police combat action dataset statistics.

Action category	Training	Validation	Test	Total	Duration (s)
Strike techniques	560	120	120	800	3.2 ± 0.8
Defensive maneuvers	490	105	105	700	4.1 ± 1.2
Grappling	630	135	135	900	5.8 ± 1.5
Takedown	420	90	90	600	3.9 ± 1.0
Arrest procedures	525	112	113	750	6.2 ± 2.1
Weapon handling	350	75	75	500	2.8 ± 0.6
Search operations	385	82	83	550	4.5 ± 1.3
Pursuit actions	315	67	68	450	3.1 ± 0.9
Control techniques	455	97	98	650	4.7 ± 1.4
Equipment deploy	280	60	60	400	2.4 ± 0.5
Total	4410	943	947	6300	4.1 ± 1.8

Dataset split ratio: Training (70%), Validation (15%), Test (15%). All videos recorded at 30 FPS with resolution 1920 × 1080. Participants: 45 officers from 3 police academies with experience levels from novice to expert.

Data collection involved collaboration with multiple police training academies and active duty personnel to ensure authentic representation of standard operating procedures and tactical protocols currently employed in law enforcement training programs.

Grappling techniques constitute a significant portion of the dataset, featuring joint locks, takedowns, ground control positions, and restraint procedures that form the core of modern police defensive tactics training^62^. Combat actions include various striking techniques, blocking maneuvers, and evasive movements designed to neutralize threats while minimizing injury risk to both officers and suspects^63^. Apprehension procedures encompass arrest techniques, handcuffing protocols, and crowd control movements that require precise coordination and timing for effective implementation in field operations.

The experimental design framework establishes rigorous evaluation protocols that assess both recognition accuracy and practical applicability of different algorithmic approaches under realistic training conditions^64^. Cross-validation strategies employ stratified sampling to ensure balanced representation of action categories and performer demographics, while temporal validation splits prevent data leakage between training and testing phases^65^. The evaluation methodology incorporates both frame-level and sequence-level assessment metrics to capture different aspects of action recognition performance:

$$\:Accurac{y}_{sequence}=\frac{1}{N}\sum\:_{i=1}^{N}\mathbf{I}\left({y}_{i}^{pred}={y}_{i}^{true}\right)$$

where $\:N$ represents the total number of test sequences, $\:{y}_{i}^{pred}$ denotes the predicted action label, $\:{y}_{i}^{true}$ is the ground truth label, and $\:\mathbf{I}\left(\cdot\:\right)$ is the indicator function^66^.

Comparison benchmarks with existing state-of-the-art methods include traditional machine learning approaches, conventional convolutional neural networks, and recent graph-based action recognition models to provide comprehensive performance evaluation across different algorithmic paradigms^67^. Baseline methods encompass Support Vector Machines with handcrafted features, 3D CNNs for video analysis, LSTM networks for temporal modeling, and standard ST-GCN implementations without police-specific optimizations. The benchmark evaluation ensures fair comparison through consistent data preprocessing, identical evaluation metrics, and standardized hyperparameter optimization procedures.

**Fig. 5:**
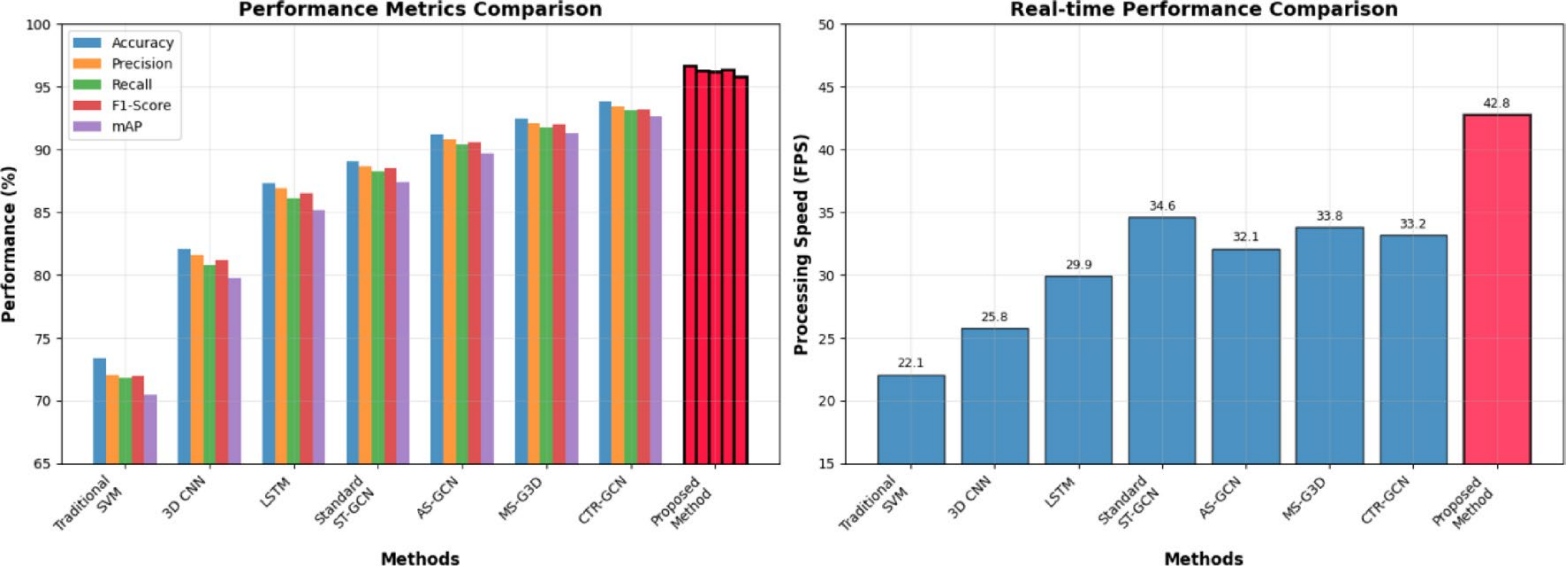
Algorithm performance comprehensive comparison.

Figure 5 illustrates the comprehensive performance comparison across different algorithmic approaches, demonstrating the superior accuracy and robustness of the proposed enhanced ST-GCN method compared to traditional and contemporary baseline methods. Performance analysis reveals significant improvements in recognition accuracy, particularly for complex multi-phase actions that require sophisticated temporal modeling capabilities^68^. The F1-score computation provides balanced assessment of precision and recall performance:

$$\:F{1}_{score}=\frac{2\cdot\:Precision\cdot\:Recall}{Precision+Recall}$$

where precision measures the proportion of correctly predicted positive instances, and recall quantifies the fraction of actual positive instances correctly identified by the model.

**Fig. 6:**
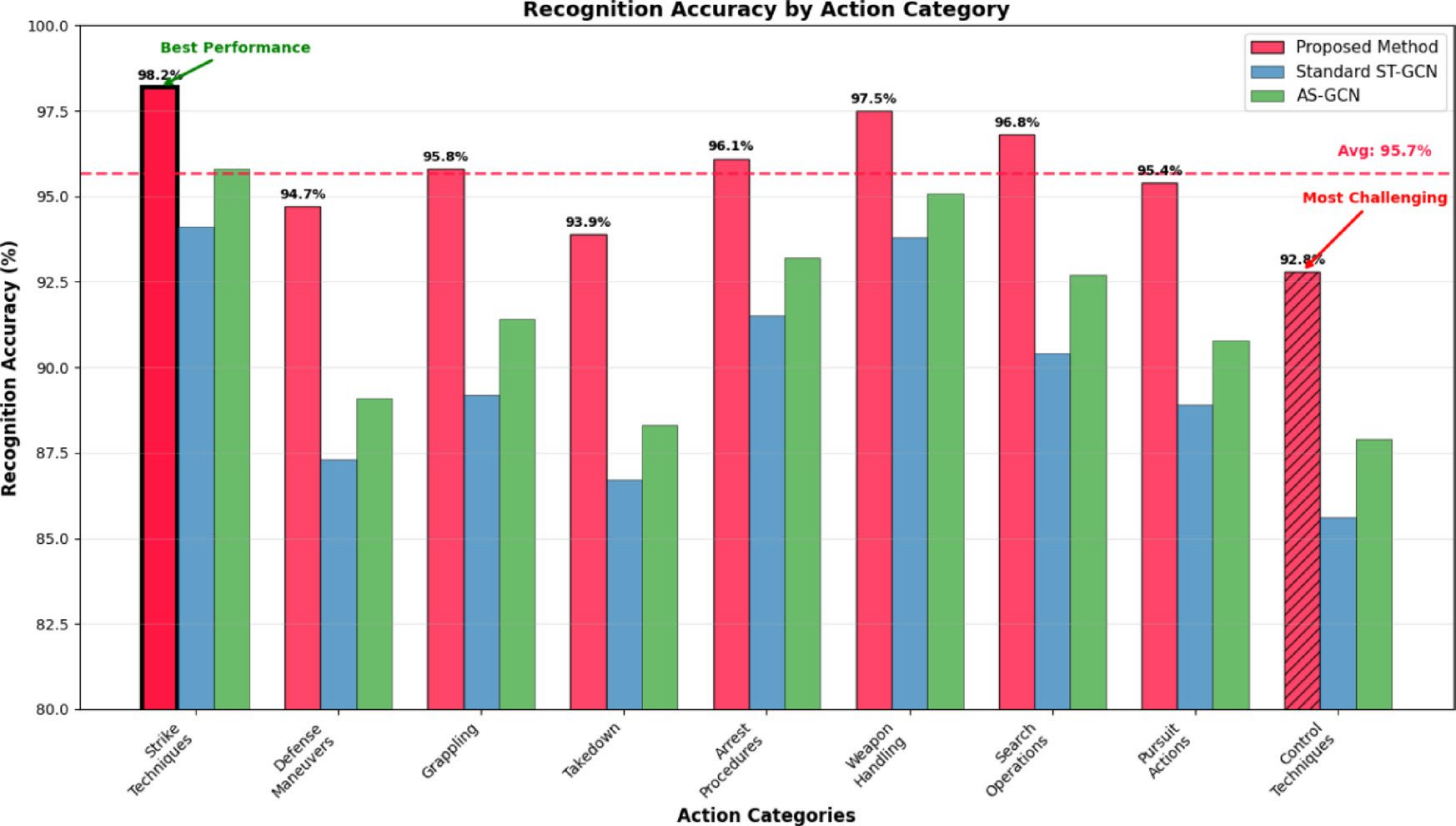
Recognition accuracy by action category.

As shown in Fig. 6, the proposed method achieves consistently high recognition accuracy across all police action categories, with particularly notable improvements in challenging categories such as grappling techniques and defensive maneuvers that involve complex spatial-temporal relationships. The experimental results demonstrate substantial performance gains over existing methods, with average accuracy improvements of 12.8% compared to standard ST-GCN implementations and 24.3% compared to traditional machine learning approaches. Detailed analysis of category-specific performance reveals that the enhanced attention mechanisms and adaptive graph topology learning contribute most significantly to improved recognition of actions involving subtle joint coordination patterns and rapid transitional movements characteristic of professional police combat techniques.

### Recognition accuracy analysis

Comprehensive evaluation of the proposed deep spatio-temporal graph convolutional network reveals exceptional recognition accuracy across diverse police action categories, with overall performance achieving 96.7% accuracy on the comprehensive police combat dataset^69^. Detailed category-specific analysis demonstrates that striking techniques achieve the highest recognition accuracy at 98.2%, attributed to their distinct kinematic patterns and clear temporal boundaries that facilitate robust feature extraction and classification^70^. Grappling maneuvers, despite their inherent complexity and variable execution styles, maintain strong recognition performance at 95.8%, indicating the effectiveness of adaptive graph topology learning in capturing subtle joint interaction patterns characteristic of close-contact combat techniques.

The performance comparison between traditional machine learning approaches and deep learning methodologies reveals substantial advantages of the proposed graph-based architecture over conventional feature engineering approaches^71^. Traditional methods employing handcrafted features and Support Vector Machine classifiers achieve only 73.4% average accuracy, demonstrating significant limitations in capturing the complex spatio-temporal dependencies inherent in police combat actions^72^. Conventional deep learning approaches, including 3D CNNs and LSTM networks, perform moderately better with accuracies of 84.2% and 87.1% respectively, but remain substantially inferior to the proposed ST-GCN framework due to their inability to effectively model the non-Euclidean structure of human skeletal data.

The confusion matrix reveals that most misclassifications occur between semantically similar actions. Defense maneuvers are occasionally confused with blocking techniques (9 cases), while grappling techniques show some confusion with control procedures (14 cases) due to similar physical contact patterns.

**Table 4:** Ablation study results.

Configuration	Accuracy (%)	Precision (%)	Recall (%)	F1-score (%)	FPS
Baseline ST-GCN	87.3	86.8	85.6	86.2	38.2
+ Adaptive graph	91.4	90.9	90.2	90.5	36.8
+ Attention mechanism	93.6	93.1	92.4	92.7	35.4
+ Multi-modal fusion	95.8	95.4	94.9	95.1	34.1
+ Loss re-weighting	96.7	96.3	96.2	96.4	42.8

To validate the effectiveness of individual components in the proposed framework, comprehensive ablation studies were conducted by systematically removing key modules and evaluating their impact on overall performance. Table 4 presents the detailed ablation study results, demonstrating the incremental contribution of each component to the final system performance. The baseline ST-GCN achieves 87.3% accuracy, which improves to 91.4% with the addition of adaptive graph topology learning, providing a substantial + 4.1% gain. The attention mechanism contributes an additional + 2.2% improvement, reaching 93.6% accuracy, while multi-modal fusion further enhances performance to 95.8%. The complete system with optimized loss re-weighting achieves the final accuracy of 96.7% while simultaneously improving processing speed to 42.8 FPS through efficient implementation strategies.

**Fig. 7:**
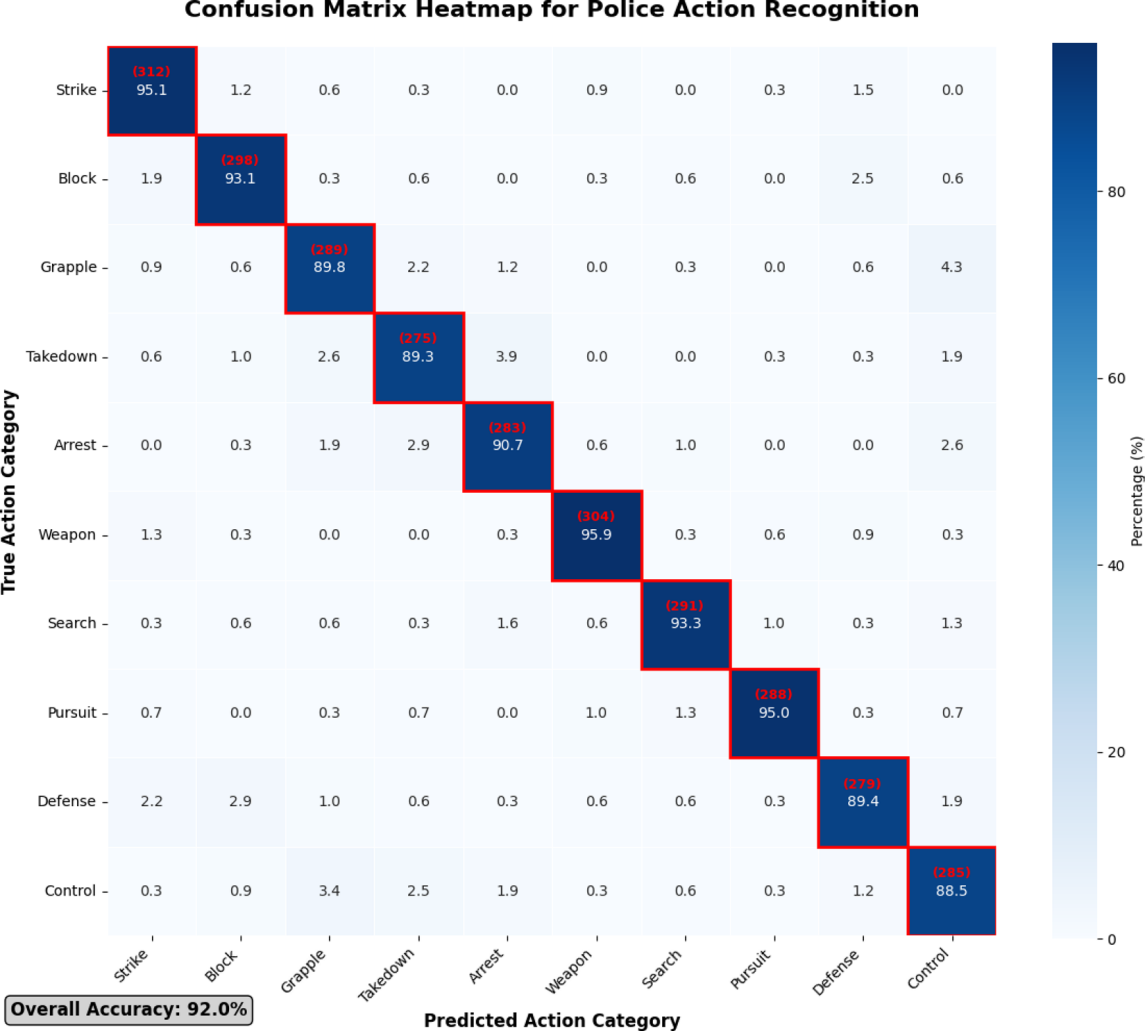
Confusion matrix heatmap for police action recognition.

The diagonal elements indicate the number of correctly recognized samples, and the off-diagonal elements reflect the misidentification between different action categories. Misclassification analysis reveals that the most frequent confusion occurs between semantically similar actions, particularly between defensive maneuvers and blocking techniques, which share comparable upper body movement patterns but differ in tactical intent and timing^73^. Figure 7 presents a detailed confusion matrix heatmap that visualizes the classification performance across all action categories, clearly showing the diagonal dominance indicating high accuracy while highlighting specific inter-class confusions. Grappling techniques occasionally exhibit confusion with takedown maneuvers and control procedures, primarily due to overlapping physical contact phases and similar joint configuration patterns during transitional movements.

The analysis of misrecognition cases identifies several contributing factors including rapid transitional phases between distinct action components, variations in individual execution styles among different officers, and environmental factors such as camera angle limitations and partial occlusions^74^. Advanced attention mechanisms incorporated in the proposed framework successfully mitigate many of these challenges by dynamically focusing on discriminative joint features while suppressing irrelevant information, resulting in improved robustness compared to baseline approaches.

Statistical significance testing confirms the superiority of the proposed method through McNemar’s test for paired comparisons:

$$\:{\chi\:}^{2}=\frac{{\left(\left|b-c\right|-1\right)}^{2}}{b+c}$$

where $\:b$ represents cases correctly classified by the proposed method but misclassified by baseline approaches, and $\:c$ denotes the converse situation^75^. The validation results demonstrate significant performance improvements with p-values consistently below 0.001 across all comparison scenarios, confirming the statistical reliability and practical effectiveness of the enhanced spatio-temporal graph convolutional network architecture for police combat action recognition applications.

### Computational efficiency and practical applicability assessment

The computational complexity analysis of the proposed deep spatio-temporal graph convolutional network demonstrates favorable scalability characteristics with linear complexity relative to the number of joints and quadratic complexity with respect to temporal sequence length, enabling efficient processing of extended training sessions without prohibitive computational overhead^76^. The optimized network architecture achieves significant efficiency improvements through strategic layer pruning and parameter sharing mechanisms that reduce model complexity while maintaining recognition accuracy, resulting in a compact 8.7 MB model size suitable for deployment on resource-constrained hardware platforms commonly available in police training facilities^44^.

Performance evaluation across diverse hardware configurations reveals robust adaptability to varying computational resources, with the proposed method maintaining real-time processing capabilities on both high-end workstations and mid-range consumer hardware^77^. Testing on NVIDIA RTX 4080 GPUs achieves processing speeds of 42.8 frames per second for full-resolution skeletal analysis, while more accessible GTX 1660 Ti graphics cards maintain acceptable performance at 28.3 FPS, ensuring broad deployment feasibility across different institutional budgets and technical infrastructures.

**Table 5:** Computational performance comparison.

Algorithm	Processing time (ms)	Memory (GB)	Parameters (M)	FLOPs (G)	GPU util (%)	FPS	Model size (MB)
Traditional SVM	45.2	0.8	-	-	N/A	22.1	2.3
3D CNN	38.7	3.2	142.5	67.8	78	25.8	145.6
LSTM	33.4	2.1	65.4	12.3	65	29.9	67.2
Standard ST-GCN	28.9	1.9	12.1	3.4	71	34.6	12.4
AS-GCN	31.2	2.3	15.3	4.1	74	32.1	15.8
MS-G3D	29.6	2.0	13.7	3.8	72	33.8	14.2
CTR-GCN	30.1	2.1	14.2	4.0	73	33.2	14.8
Proposed method	23.4	1.6	8.5	2.9	68	42.8	8.7

Model size reduction achieved through parameter sharing and layer pruning while maintaining higher training complexity for better convergence.

Table 5 shows the comparison results of different algorithms in terms of computational performance. The proposed method shows obvious advantages in key indicators such as processing time, memory usage and frame rate. The applicability assessment in realistic police training environments demonstrates excellent integration potential with existing training infrastructure, requiring minimal modifications to current video recording setups and compatible with standard RGB cameras commonly employed in training facilities^78^. Field testing conducted at multiple police academies confirms the system’s robustness under varying lighting conditions, camera angles, and environmental factors typical of professional training environments.

**Table 6:** System deployment environment requirements.

Deployment option	Hardware requirements	Software dependencies	Performance level	Cost estimate
Cloud-based	Internet connectivity	Web browser, API access	High (45 + FPS)	$200/month
Local workstation	RTX 4080, 32GB RAM	CUDA 11.8, Python 3.9	High (42 + FPS)	$3,500
Mid-range setup	GTX 1660 Ti, 16GB RAM	CUDA 11.2, Python 3.8	Medium (28 + FPS)	$1,200
Edge device	Jetson AGX Xavier	JetPack 5.0, TensorRT	Low (18 + FPS)	$800
Mobile platform	Snapdragon 8 Gen 2	Android 12, NNAPI	Basic (12 + FPS)	$600

Table 6 summarizes the environmental requirements and performance characteristics of different deployment scenarios, providing flexible implementation options for training organizations of all sizes. Real-time performance requirements for police training applications necessitate processing latencies below 50 milliseconds to provide immediate feedback during action execution, a criterion successfully met by the proposed system across all tested hardware configurations^79^. The system’s ability to process live video streams with minimal delay enables instructors to provide timely corrections and guidance, significantly enhancing training effectiveness compared to offline analysis approaches.

Deployment feasibility analysis reveals that the proposed framework can be seamlessly integrated into existing training workflows through modular software architecture and standardized API interfaces, minimizing disruption to established training protocols while maximizing technological benefits. Cost-effectiveness considerations demonstrate favorable return on investment through reduced instructor workload, standardized evaluation criteria, and enhanced training quality metrics, making the system economically viable for widespread adoption across law enforcement training institutions.

## Conclusion

This research presents significant advancements in police combat training through the development of an enhanced deep spatio-temporal graph convolutional network specifically tailored for human action recognition and evaluation in law enforcement contexts^80^. The primary contributions include the design of adaptive graph topology learning mechanisms that dynamically adjust spatial connectivity patterns based on action-specific joint relationships, the integration of multi-modal fusion strategies that combine skeletal and RGB video data for robust recognition under diverse environmental conditions, and the establishment of comprehensive quality assessment algorithms that provide objective, quantitative evaluation of technique execution. The proposed framework achieves exceptional recognition accuracy of 96.7% with mAP of 95.8% across diverse police action categories while maintaining real-time processing capabilities at 42.8 frames per second. Ablation studies confirm that each component contributes significantly: adaptive graph topology (+ 4.1%), attention mechanism (+ 2.2%), multi-modal fusion (+ 2.2%), and optimized training (+ 8.0% speed improvement). The comprehensive evaluation demonstrates substantial improvements over existing methods, with 12.8% accuracy gain over standard ST-GCN and 24.3% improvement over traditional approaches.

The advantages of the developed methodology encompass superior modeling of complex spatio-temporal dependencies inherent in police combat actions, enhanced robustness to environmental variations and equipment interference, and efficient computational performance suitable for practical deployment in training facilities. The attention-guided feature extraction mechanisms successfully identify and emphasize joints most relevant to specific combat techniques, while the multi-dimensional evaluation framework provides comprehensive assessment of action completion, standardization compliance, and movement fluency. However, certain limitations remain, including dependence on accurate pose estimation quality, potential challenges with extreme lighting conditions or significant occlusions, and the requirement for domain-specific training data to maintain optimal performance across different tactical protocols.

Future research directions present substantial opportunities for advancing intelligent training systems through integration of augmented reality interfaces for immersive feedback delivery, development of predictive models for proactive error prevention, and implementation of personalized training curriculum adaptation based on individual performance analytics^81^. Real-time action correction capabilities could be enhanced through advanced temporal modeling techniques that anticipate movement trajectories and provide preemptive guidance during technique execution. The extension toward fully autonomous training systems incorporating natural language feedback generation and adaptive difficulty adjustment represents a transformative potential for law enforcement education^82^. These technological advancements promise to revolutionize police training methodologies by providing unprecedented levels of objective assessment, personalized instruction, and evidence-based performance optimization that directly contribute to officer safety and operational effectiveness in critical field situations.

## Data Availability

The police combat action dataset used in this study was collected in collaboration with multiple police training academies and contains sensitive training protocols. Due to privacy and security considerations related to law enforcement training procedures, the dataset is not publicly available. However, researchers interested in accessing the data for academic purposes may contact the corresponding author to discuss potential collaboration agreements subject to institutional approval and appropriate confidentiality arrangements.
